# Cloning the *hbs* gene from *Bacillus subtilis* and expression of the HBsu protein in *Escherichia coli*

**Published:** 2010-09

**Authors:** S Ghodsi, S Gharavi, P Ghadam

**Affiliations:** Biology Department, Faculty of Sciences, Alzahra University, Vanak, Tehran, IR Iran

**Keywords:** *Bacillus subtilis*, hbs gene, cloning, expression

## Abstract

**Background and Objectives:**

*Bacillus subtilis* HBsu is a 10 kD heat-stable protein shown to be involved in binding to DNA and is encoded by the *hbs* gene. Large–scale production for biochemical analysis is achieved through cloning and expression of the recombinant protein.

**Materials and Methods:**

This gene was amplified from *B. subtilis* ATCC 6633 using PCR and cloned into pET28a (+) expression vector. The construct was used to transform *Escherichia coli* BL21 (DE3). The expression of the protein was induced by the addition of 1mM IPTG. To confirm the expression of the cloned gene, SDS-PAGE was carried out and production of an approximately 11 KD recombinant tagged protein was confirmed for the cloned *hbs* gene.

**Results and Conclusion:**

The identity of the recombinant HBsu was verified and characterized by SDS-PAGE which can then be utilized for further applications.

## INTRODUCTION

In prokaryotes, a number of abundant, small, basic, and heat-stable proteins have been identified which wrap DNA without obvious sequence specificity. Among bacteria, the primary structures of these proteins are highly conserved and have been designated as histone-like proteins (HLPs) (1). One of the best-studied HLPs is HU of *Escherichia coli* (2). In *E.coli*, HU is a heterodimer composed of two highly homologous subunits of ∼9 kD each, whereas in many other bacteria, HU is present as a homodimer (3, 4). HU is a very conserved protein in the prokaryotic world (4, 5). HU is also present in chloroplasts (5) as well as in an eukaryotic virus (5, 6). *In vivo*, HU was shown to contribute to the maintenance of DNA superhelical density and to modulate topoisomerase I activity (5, 9). HU plays a role in the initiation of oriC-dependent DNA replication (3, 8), DNA recombination (5, 9), Mu transposition (10–12) and transcriptional regulation (1, 13). Cells lacking HU are extremely sensitive to γ and UV irradiation (14, 15).

The *Bacillus subtilis* genome encodes for one histone-like protein by the *hbs* gene (16, 17). HBsu is the homolog of the HU proteins of *E. coli* (17, 18) and as in HU; HBsu binds DNA non specifically (19). DNA binding by HBsu is independent of cofactors or additional proteins (17) and furthermore, HBsu enables β-recombinase-mediated recombination by stabilizing a DNA secondary structure (20).

For further insight into the function of HBsu in other microorganisms, purified HBsu for antiserum preparation is essential. So this study was designed to clone the *hbs* gene from *B. subtilis* and express the recombinant clone in *E. coli* with the aim of high level HBsu protein production in the heterologous host.

## MATERIALS AND METHODS

**Bacterial strains and growth conditions.** *Bacillus subtilis* ATCC 6633 was grown on nutrient agar at 28°C for 24 h. *E. coli* DH5α and *E. coli* BL21 (DE3) were cultured on Luria-Bertani medium overnight at 37°C. Following transformation, *E.coli* colonies carrying the recombinant vector were selected on LB medium with 50µg/µl kanamycin.

**Nucleic acid preparation.** Genomic DNA from *Bacillus subtilis* ATCC 6633 was extracted by the phenol-chloroform method.

**Cloning of *B. subtilis hbs* gene in pET28a (+).** The primers EcoR 5′ hbs(CAGTGAATTCATGAACAAAACAGAACTTACT) and Hind 3′ hbs (GATGAAGCTTTTATTTTCCGGCAACTGC) were selected based on the *B. subtilis* ATCC 23857 *hbs* gene sequence in the Gene Bank. The primers included an *Eco* R′ restriction site at the 5′ end of the gene and a *Hind* III restriction site at the 3′ end. The *hbs* gene was amplified from *B. subtilis* by PCR in a reaction containing 0.01 µg/µl template DNA, 0.5 µM of each primer, 5 µl of 10X *Taq* polymerase buffer, 2 mM of MgCl_2_, 0.2 mM of each dNTP, 0.5 µl of Taq polymerase in 50 µl volume. Amplification was performed in a thermal cycler (PEQlab) and initiated with a primary denaturation step at 94°C for 5 min, followed by 35 cycles of 94°C for 30 sec, 54°C for 30 sec and 72°C for 20 sec and 5 min for final extension. PCR product was separated on 2% agarose gel and visualized by ethidium bromide staining. Following the initial confirmation, the amplicon was purified with DNA extraction kit (Fermentas, Lithuania), after which it was digested with *Eco*R′ and *Hind* III (Fermentas, Lithuania). The same digestion reaction was carried out on pET28a (+) (Novagen). Ligation (overnight, at room temperature) was done with T4 Ligase (Fermentas, Lithuania) after which the resulting plasmid containing *hbs* gene sequence, was used to transform *E. coli* (DH5α). All reactions such as digestion, ligation and transformation procedures were performed according to the manufacturer's instructions.

**Cloning confirmation.** To confirm the presence of the recombinant plasmid in the transformed cells, the plasmid was extracted from the cells by Plasmid Mini Extraction Kit (Bioneer, South Korea) and analyzed by PCR. The PCR product was sequenced and the sequence compared with its Gene Bank origin for confirmation.

**Expression of the recombinant *hbs* gene.** The recombinant plasmids containing the *hbs* gene sequence, were used to transform *E.coli* Bl21( DE3). To assess the expression of *hbs*, positive colonies were cultured in LB medium containing 50µg/µl kanamycin following which 1 mM IPTG (Isopropyl-beta-D-thiogalacto-pyranoside) was added as an inducer to the medium with OD=0.6 and samples were collected before induction and 1, 2, 3, 4, 6 and 12 hrs after induction. The cells were harvested, treated with lysis buffer (sodium chloride 300 mM, sodium phosphate 50 mM and imidazole 10 mM), centrifuged and the supernatant used for SDS-PAGE analysis. Gels were stained with Coomassie Blue R250 and the quantity of the expressed protein was estimated by comparing the intensity of the protein bands.

**Purification of the cell mass.** Cells harvested from production medium, were lysed and the recombinant HBsu protein purified by Ni-NTA column as specified by the manufacturer's instructions (Novagen). The purified protein was subsequently analyzed by SDS-PAGE.

## RESULTS

**Fig. 1:**
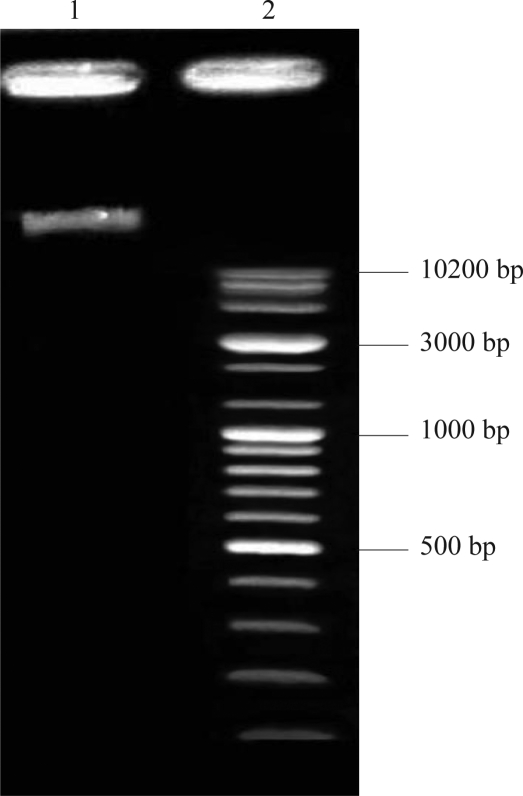
Extracted Genomic DNA from *Basillus subtilis* ATCC 6633 by phenol-chloroform method. Lane 1: Extracted genomic DNA. Lane 2: 10kb DNA ladder.

**Fig. 2:**
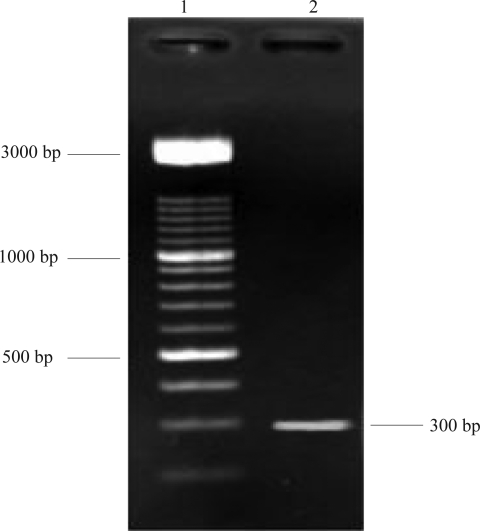
Amplification of *hbs* gene by PCR. Lane 1: 3kb DNA ladder. Lane 2: *hbs* gene PCR product.

Cloning of the *hbs* gene. Genomic DNA from *Bacillus subtilis* ATCC 6633 was extracted by phenol-chloroform method (Fig. 1) and the *hbs* gene was amplified by PCR using primers EcoR5′ and Hind 3′. The resulting product had a size of approximately 300 bp which was the expected size of the gene (Fig. 2).

The PCR product was electrophoresed on a 2% agarose gel and the band purified by DNA Extraction Kit. For the insertion of the gene in the vector, recognition site for *EcoR′* and *Hind III* were introduced on the EcoR 5′ and Hind 3′ ends, respectively. The same recognition sequences on the polylinker site of the pET28a (+) made the ligation reaction possible in the correct direction.

**Fig. 3:**
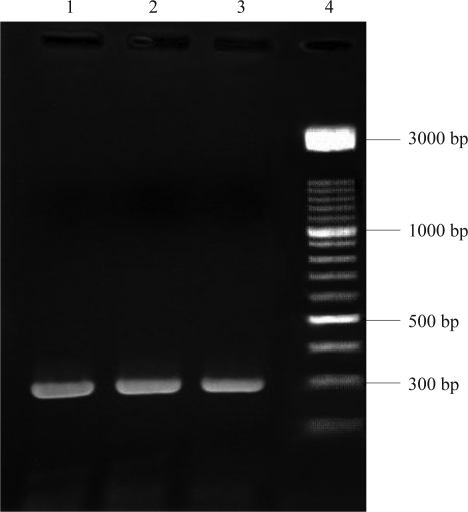
Amplification of *hbs* gene by PCR on the vector. Lane 1, 2, 3: *hbs* gene PCR product. Lane 4: 3kb DNA ladder

A PCR reaction was performed with EcoR 5′ and Hind 3′ primers on the vector, on the outer borders of the inserted sequence. The PCR product has a size of approximately 300bp (Fig. 3).

The cloned *hbs* was sequenced (Macrogene, South Korea) to verify the identity of the clone. The sequence of the gene was identical to the sequence deposited in the Gene Bank.

**Fig. 4:**
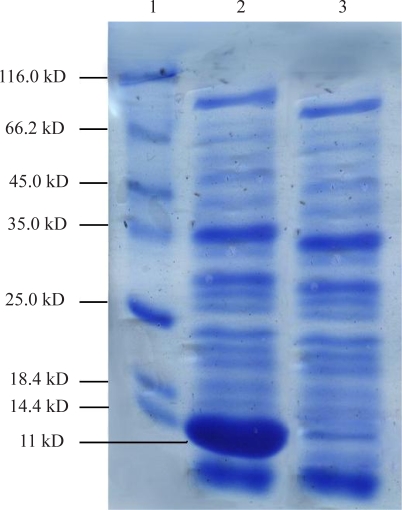
15% SDS-PAGE. Lane 1: Protein molecular size marker. Lane 2: Lysate of *E.coli* cells containing recombinant vector, collected 12 h after induction. Lane 3: Lysate of *E. coli* cells containing recombinant vector, collected before induction

**Expression of *B.* subtilis HBsu protein.** The recombinant protein expression was induced by the addition of 1 mM IPTG as an inducer. The optimum incubation time after addition of IPTG was determined to be 12h. The expressed protein was an intra cellular protein and hence detected only in the cell lysate. SDS-PAGE analysis shows a novel band of approximately 11KD (Fig. 4).

This band matches with the expected molecular weight for recombinant *B. subtilis* HBsu protein. This protein is a 10KD protein, but in the recombinant form as a result of fusion fragment of six-histidine in the N-terminal has a molecular weight of 11 KD. This tag was used for purification by Ni-NTA affinity column.

**Fig. 5:**
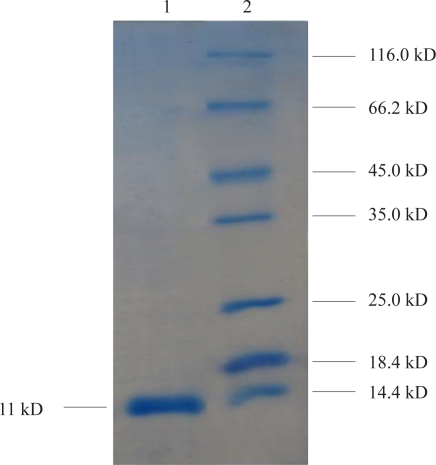
The recombinant HBsu protein was purified in *E.coli*. Lane 1: Purified HBsu protein. Lane 2: Protein molecular size marker

**Purification of the recombinant protein.** Cell mass was harvested and subjected to purification procedure as specified by manufacture's instruction of Ni-NTA affinity column. The expected band was obtained in eluted fraction (Fig. 5).

## DISCUSSION

More than 30 members of the family of histone-like proteins, all with a size of about 90 amino acids and an overall basic net charge, have been identified (4). One of them is the HU family. Several proteins with properties and structures highly homologous to those of HU protein have been isolated from bacteria and bacteriophage but it seems that HU protein sequence from various strains of one species could differ. Amongst some *Bacillus* species, a protein highly homologous to HU, classified as HB has been isolated and characterized (17). Among the HB proteins of different *Bacillus* species so far sequenced, conservation is more than 80% (21). *B. subtilis* genome encodes one HB protein by *hbs* gene which is known as the HBsu (22).

The *hbs* gene has been cloned and expressed by many investigators and properties of recombinant protein investigated. Micka *et al*., (16) have cloned, sequenced and characterized the *B.subtilis* gene encoding the DNA-binding protein HBsu while Groch *et al*. (23) expressed a synthetic gene encoding the histone-like DNA binding protein HBsu from *B. subtilis* in *E. coli* and compared it with wild-type protein. Kohler *et al.,* (19) constructed a *hbs*-GFP fusion to investigate the physiological role of the essential histone-like protein of *B.subtilis* (HBsu) in nucleoid structureLater, Ross and Setlow (18) cloned and expressed *hbs* gene to investigate the levels of HBsu in the spore and the localization of the HBsu in the forespore.

In this study, we have successfully cloned and expressed *B.subtilis hbs* gene and purified HBsu protein. pET 28a (+) is a vector which permits high expression and rapid purification of the recombinant protein through a his-tag fused to the expressed protein at N-terminus High level expression of the recombinant HBsu protein facilitates the production of large amounts of the desired protein required for further studies.

## References

[1] Micka B, Groch N, Heinemann U, Marahiel MA (1991). Molecular cloning, nucleotide sequence and characterization of the *Bacillus subtilis* gene encoding the DNA-binding protein Hbsu. J Bacteriol.

[2] Nash HA, Lin ECC, Lynch AS (1996). The HU and IHF proteins: Accessory factors for complex protein-DNA assemblies. R. G. Landes Company.

[3] Kamashev D, Rouviere-Yaniv J (2000). The histone-like protein HU binds specifically to DNA recombination and repair intermediates. EMBO J.

[4] Oberto J, Rouviere-Yaniv J (1996). Serratia marcescens contains a heterodimeric HU protein like *Escherichia coli* and *Salmonella typhimurium*. J Bacteriol.

[5] Kamashev D, Balandina A, Mazur AK, Arimondo PB, Rouviere-Yaniv J (2008). HU bind and folds single-stranded DNA. Nucleic Acid Res.

[6] Neilan JG, Lu Z, Kutish GF, Sussman MD, Roberts PC, Yozawa T, Rock DL (1993). An African swine fever virus gene with similarity to bacterial DNA binding proteins, bacterial integration host factors, and *Bacillus* phase SPO1 transcription factor, TF1. Nucleic Acid Res.

[7] Bensaid A, Almeida A, Drlica K, Rouviere-Yaniv J (1996). Cross-talk between topoisomerase I and HU in *Escherichia coli*. Mol Biol.

[8] Ryan VT, Grimwade JE, Nievera CJ, Leonard AC (2002). IHF and HU stimulate assembly of pre-replication complexes at *Escherichia coli* oriC by two different mechanisms. Mol Biol.

[9] Dri AM, Moreau P, Rouviere-Yaniv J (1992). Involvement of the histone-like proteins OsmZ and HU in homologous recombination. Gene.

[10] Kobryn K, Lavoie BD, Chaconas G (1999). Supercoiling-dependent site-specific binding of HU to naked Mu DNA. Mol Biol.

[11] Lavoie BD, Chaconas G (1993). site-specific HU binding in the MU transposon: Conversion of a sequence-independent DNA-binding protein into chemical nuclease. Genes Dev.

[12] Lavoie BD, Shaw G, Millner A, Chaconas G (1996). Anatomy of a flexer-DNA complex inside a higher-order transposition intermediate. Cell.

[13] Aki T, Dhya SA (1997). Repressor induced site-specific binding of HU for transcriptional regulation. EMBO J.

[14] Boubrik F, Rouviere-Yaniv J (1995). Increased sensitivity to γ irradiation in bacteria lacking protein HU. Proc Natl Acad Sci USA.

[15] Li S, Waters R (1998). *Escherichia coli* strains lacking HU are UV sensitive due to a role for HU in homologous recombination. J Bacteriol.

[16] Micka B, Marahiel MA (1992). The DNA-binding Hubs is essential for normal growth and development in *Bacillus subtilis*. Biochimie.

[17] Wolfgang K, Marahiel MA (2002). Structure- function relationship and regulation of two *Bacillus subtilis* DNA-binding protein, HBsu and AbrB. J Mol Microbiol Biotechnol..

[18] Ross MA, Setlow P (2000). The Bacillus subtilis HBsu protein modifies the effects of alpha/beta-type, small acid-soluble spore proteins on DNA. J Bacteriol.

[19] Kohler P, Marahiel MA (1997). Association of the histone-like protein hubs with nucleotide of *Bacillus subtilis*. J Bacteriol.

[20] Alonso JC, Gutierrez C, Rojo F (1995). The role of chromatin-associated protein Hbsu in beta-mediated DNA recombination is to facilitate the joining of distant recombination sites. Mol Biol.

[21] Kamau E, Tsihlis ND, Simmons LA, Grove A (2005). Surface salt bridges modulate the DNA site size of bacterial histone-like HU proteins. Biochem J.

[22] Kunst F, Ogasawara N, Moszer I, Albertini AM, Alloni G (1997). The complete genome sequence of the gram-positive bacterium *Bacillus subtilis*. Nature.

[23] Groch N, Schindein H, Scholtz AS, Hahn U, Heinemann U (1992). Determination of DNA-binding parameters for the *Bacillus subtilis* histone-like HBsu protein through introduction of fluorophores by site-directed mutagenesis of a synthetic gene. J Biochem.

